# Advances in the Application of Bi-Based Compounds in Photocatalytic Reduction of CO_2_

**DOI:** 10.3390/molecules28103982

**Published:** 2023-05-09

**Authors:** Cheng Zuo, Qian Su, Zaiyong Jiang

**Affiliations:** College of Chemistry & Chemical and Environmental Engineering, Weifang University, Weifang 261061, Chinasqian316@wfu.edu.cn (Q.S.)

**Keywords:** CO_2_ reduction, Bi-based catalysts, photocatalysis, heterostructure, vacancies

## Abstract

Bi-based semiconductor materials have special layered structure and appropriate band gap, which endow them with excellent visible light response ability and stable photochemical characteristics. As a new type of environment-friendly photocatalyst, they have received extensive attention in the fields of environmental remediation and energy crisis resolution and have become a research hotspot in recent years. However, there are still some urgent issues that need to be addressed in the practical large-scale application of Bi-based photocatalysts, such as the high recombination rate of photogenerated carriers, limited response range to visible spectra, poor photocatalytic activity, and weak reduction ability. In this paper, the reaction conditions and mechanism of photocatalytic reduction of CO_2_ and the typical characteristics of Bi-based semiconductor materials are introduced. On this basis, the research progress and application results of Bi-based photocatalysts in the field of reducing CO_2_, including vacancy introduction, morphological control, heterojunction construction, and co-catalyst loading, are emphasized. Finally, the future prospects of Bi-based photocatalysts are prospected, and it is pointed out that future research directions should be focused on improving the selectivity and stability of catalysts, deeply exploring reaction mechanisms, and meeting industrial production requirements.

## 1. Introduction

With the rapid development of the economy, a large amount of fossil fuels are consumed. It leads to the release of a large amount of carbon dioxide, causing the greenhouse effect and thus breaking the world’s ecological balance. Additionally. fossil fuels are also non-renewable energy [1]. The concentration of carbon dioxide increases is seriously impacting human habitats and the earth’s ecosystem. Therefore, it has become one of the most important research topics in the world to explore how to reduce the amount of CO_2_ in the atmosphere and use it rationally. Photocatalysis technology offers an excellent solution for the conversion of CO_2_ into important chemical fuels such as CH_4_ [2]. At the beginning of photocatalysis research, researchers used TiO_2_ electrodes to decompose water under visible light to produce hydrogen, thus triggering a great deal of interest in the direction of photocatalysis [3]. Therefore, it is essential to use photocatalysis to reduce CO_2_ in the atmosphere and collect and store solar energy in chemical fuels. Currently, the catalysts used in photocatalytic CO_2_ reduction, such as noble metals (Pt and Au) [3,4], non-precious metals (Cu, Fe, Ni, and g-C_3_N_4_) [5,6,7,8], metal oxides (e.g., TiO_2_ and Ga_2_O_3_) [3,9,10], metal sulfides (e.g., CdS and MoS_2_) [11,12], and graphene [1,13,14] have been discovered. The successful conversion of CO_2_ into CO, CH_3_OH, CH_4,_ and other available chemicals has been reported in the literature [15,16,17,18,19]. However, the photocatalytic efficiency of most semiconductors is relatively low and not can be applied industrially. The main reasons are as follows [18,20]: (1) the separation and migration efficiency of photo-generated carriers is low, and most of the photo-generated electrons and holes will be recombined in the process of migration to the semiconductor surface, which dramatically reduces the photocatalytic performance; (2) the reaction active site is insufficient, and due to the low specific surface area, only a tiny amount of photo-generated carriers can reach the catalyst surface to react with a small amount of adsorbed CO_2_ on the surface; (3) in general, the catalytic activity of precious metals (such as Pt, Ag) is higher than that of non-precious or non-metallic metals, while the cost is high. This hinders the industrialization of photocatalysis. In this respect, the rational design of an efficient photocatalyst is necessary for the practical application of photocatalytic CO_2_ reduction.

Bi-based catalysts are widely used for photocatalytic CO_2_ reduction with the advantages of low price, excellent photoelectric activity, and environmental friendliness. For example, bismuth halide oxide (BiOX, X = Cl, Br, I) has been extensively studied in photocatalysis due to its excellent photocatalytic properties. The layered structure of BiOX provides enough space to polarize the associated atoms and orbitals, which excites the formation of an internal electric field between [Bi_2_O_2_] and halogen. The internal electric field could accelerate the separation and migration of photoexcited electron-hole pairs, and the photocatalytic activity of BiOX is significantly improved [21,22,23,24,25]. To improve the activity of Bi-based catalysts for photocatalytic reduction of CO_2_, researchers have modified the catalyst morphology, heterojunction structure, loaded co-catalysts, and introduced vacancies. Among them, introducing vacancies on the catalyst surface is an effective way to improve photocatalytic efficiency. Vacancies could effectively change the charge distribution and electron energy level of the catalyst metal elements, thus creating a synergistic effect between the elements, promoting photo-generated charge separation, and generating enough photo-generated electrons for the photocatalytic reduction of CO_2_ [26,27,28,29,30].

To our knowledge, although the evaluation of Bi-based materials continues to emerge, there are few review articles on Bi-based materials applied in photocatalytic CO_2_ reduction. Therefore, this paper systematically summarizes the recent progress of catalysts used for photocatalytic CO_2_ reduction: firstly, the basic principles of photocatalytic CO_2_ reduction are briefly introduced; secondly, the preparation methods of catalysts are introduced, focusing on the summary of the improvement strategies of photocatalytic CO_2_ reduction performance; then, several bismuth-based photocatalysts for CO_2_ reduction are introduced. Finally, the prospects and challenges of photocatalysts in CO_2_ reduction are presented.

## 2. Photocatalytic CO_2_ Reduction Foundation

### 2.1. Basic Principles of Photocatalysis

With the massive consumption of oil and fossil energy sources, CO_2_ emissions continue to rise. It has caused severe harm to the future human living environment and the earth’s ecosystem. For this reason, it has become the focus of current research to explore how to effectively reduce the amount of CO_2_ in the atmosphere and utilize it wisely. On the other hand, The resource utilization of CO_2_ requires the activation of CO_2_ molecules. In contrast, the chemical properties of CO_2_ were highly stable. The conventional CO_2_ resource utilization technology requires very high energy input to activate and convert it, which has the disadvantages of high economic cost, high energy consumption, long conversion period, and harsh reaction conditions [31]. Therefore, exploring an economical, practical, and simple CO_2_ conversion method to alleviate carbon emissions and energy shortage is strategically essential. Photocatalytic CO_2_ reduction is a relatively clean, economical, and efficient method.

**Figure 1:**
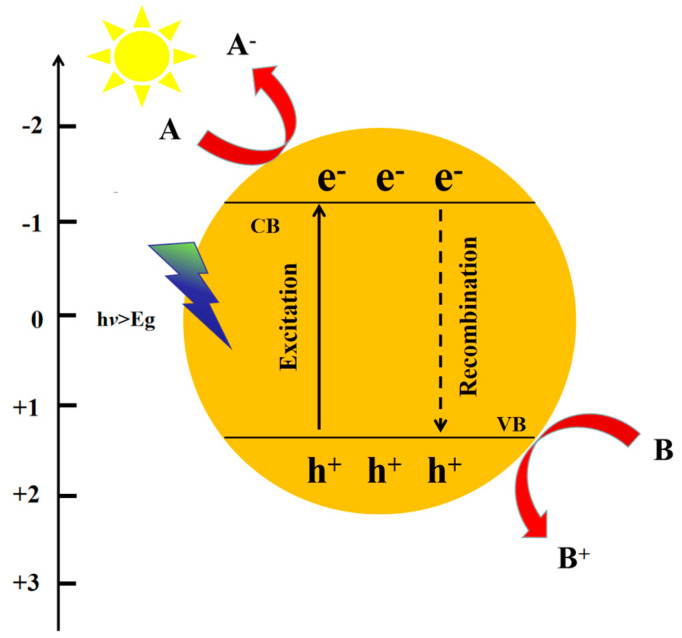
Mechanism of photocatalytic process.

With inexhaustible sunlight as the only input source and the use of suitable catalysts, photocatalytic CO_2_ reduction is possible. In the photocatalytic process (Figure 1), the absorbed photons could stimulate semiconductor (SC) photocatalysts to produce electron-hole pairs. The internal electrons will be transferred from the valence band (VB) to the conduction band (CB) inside the semiconductor after absorbing photon energy. Negatively charged electrons will be produced at the CB, and positively charged holes will be produced at the VB. Subsequently, the light-induced *e^−^*-*h^+^* will separate and migrate to the surface of the photocatalyst [32,33,34,35,36].

### 2.2. Mechanism of Photocatalytic CO_2_ Reduction

Thermodynamically, CO_2_ is one of the most stable linear molecules. The carbon atom in CO_2_ shows the highest oxidation state; the corresponding C = O bond energy is up to 750 kJ·mol^−1^; and the gap between the highest occupied molecular orbital (HOMO) and the lowest unoccupied molecular orbital (LUMO) is large (13.7 eV). The Gibbs free energy △G^0^ is −394.4 kJ·mol^−1^ [37]. Therefore, activating CO_2_ molecules requires a high input of energy. In addition, due to the optical inertness of CO_2_ in the UV visible spectral range, the photocatalytic reduction of CO_2_ requires the involvement of catalysts.

**Figure 2:**
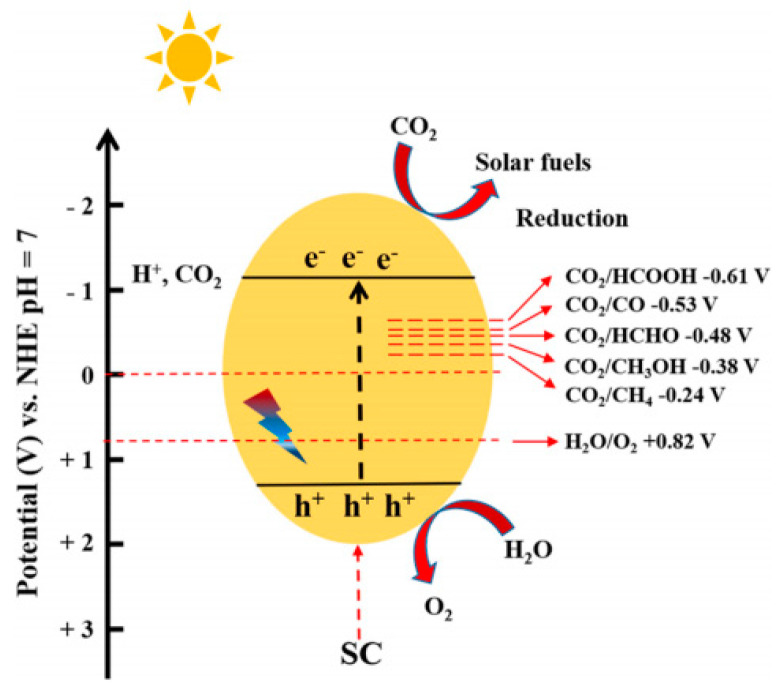
Mechanism of photocatalytic CO_2_ reduction [33]. Copyright 2022, Elsevier.

The photocatalytic reduction of CO_2_ is a process that mimics the photosynthesis of plants to convert CO_2_ into a series of hydrocarbons such as methanol, methane, and derivatives. To reduce dependence on oil and fossil fuels while reducing atmospheric CO_2_ concentrations, photocatalytic CO_2_ reduction is an excellent clean and environmentally friendly method. Photocatalytic CO_2_ reduction goes through the following steps (Figure 2): (1) CO_2_ molecules are chemically adsorbed on the surface of the photocatalyst; (2) the electrons of the semiconductor photocatalyst are activated by light with a specific wavelength and migrate from VB to CB, leaving photo-generated holes (*h^+^*) in VB; (3) photo-generated electrons (*e*^−^) and *h^+^* migrate to the catalyst surface, respectively; (4) *e*^−^ are used to activate CO_2_ and reduce it to solar fuel, while *h^+^* involved in the oxidation of H_2_O is consumed [38]. Therefore, from the perspective of photocatalytic reaction steps, an efficient photocatalyst should have the following characteristics: (1) a large specific surface area to increase the active surface sites and improve the adsorption capacity of CO_2_; (2) a narrow bandgap and appropriate band position improve solar energy utilization efficiency; (3) a nanostructure that promotes electron migration and photo-generated carrier separation is necessary; (4) rich surface defects (such as vacancies) alter the electronic and chemical properties of semiconductor, promoting the adsorption and activation of CO_2_. In addition, co-catalysts are usually added to photocatalysts to promote the separation and migration of photo-generated charge carriers and effectively reduce the reaction energy barrier for CO_2_ activation and reduction [39,40].

The photocatalytic CO_2_ reduction reaction is a proton-coupled multi-electron transfer process, and the reduction products vary with the number of electrons participating in the reaction. Two to eight electrons and corresponding numbers of protons participate in the reaction to generate HCOOH, CO, HCHO, CH_3_OH, and CH_4_, respectively. The different reduction products and corresponding electrode potentials obtained from the photocatalytic reduction of CO_2_ in an aqueous solution are shown in Table 1 [41]. Therefore, CO_2_ can be reduced to hydrocarbon fuel only when the semiconductor’s CB and VB positions meet the photocatalytic reaction’s thermodynamic requirements. Specifically, the CB position must be more negative than the CO_2_ reduction potential to transfer electrons from the semiconductor to the surface-adsorbed CO_2_. Furthermore, VB position is more positive than the H_2_O oxidation reaction. Only in this way can the reaction process of photocatalytic reduction of CO_2_ be achieved.

### 2.3. Bi-Based Catalysts

**Figure 3:**
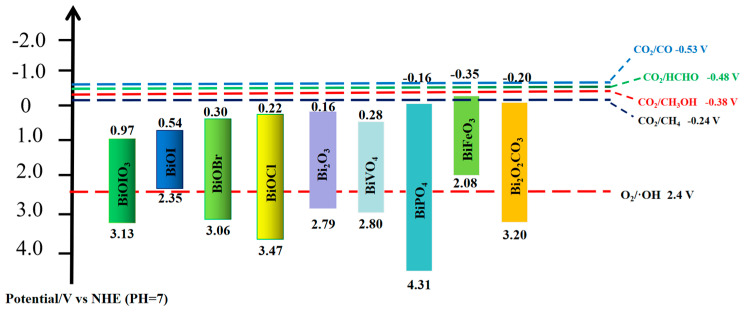
The bandgap structure of common Bi-based catalysts.

Bismuth-based catalysts have excellent electronic structure and unique redox properties. They could significantly improve the photocatalytic efficiency and the yield of products. Experience shows that the width of the band gap will affect the migration and recombination of electrons. Catalysts with wide band gaps can affect the absorption of light energy with longer wavelengths and have low utilization rates of light energy. Narrow band gap catalysts increase the probability of electron migration and recombination, reducing photocatalytic efficiency [42,43,44,45]. The band gap structures of common bismuth-based catalysts are shown in Figure 3. Most bismuth-based catalysts have a band gap of less than 3.0 eV, which means that they could be excited by solar irradiation [46,47,48]. Meanwhile, since the CB values of most bismuth-based catalysts are not negative enough, the photoinduced electrons do not have enough reduction capacity to initiate the photocatalytic reaction. The band gap of most bismuth-based catalysts satisfies the band potential energy, and the construction of heterostructures with other semiconductors could promote photo-generated electron transfer and accumulate enough electrons to participate in the photocatalytic reduction of CO_2_. Therefore, constructing intercalation structures between bismuth-based and other catalysts is a feasible way to improve photocatalytic performance [49,50].

## 3. Optimization Strategy of Bi-Based Photocatalyst

Photocatalytic CO_2_ reduction promotes the production of energy-rich hydrocarbon fuels (CO, CH_4_, and CH_3_OH) and alleviates environmental problems. Several studies have shown that atmospheric CO_2_ will increase dramatically to 550 PPM by 2100. The development of the effective surface area and potential photocatalysts on exposed crystal surfaces effectively prevents the adsorption, activation, and reduction of CO_2_ molecules.

### 3.1. Introduction of Vacancies

Vacancies or defects play a crucial role in photocatalytic applications. They could effectively change the charge distribution and electron energy level of catalysts, improving the catalyst activity. In particular, vacancies introduced in the z-structure contribute to effective interfacial interactions, thus creating a synergistic effect between elements, promoting photo-generated charge separation, and generating sufficient photo-generated electrons for the photocatalytic reduction of CO_2_. The modification effect of introducing vacancies in semiconductor catalysts is generally reflected in three aspects [51,52]: (1) the generated vacancies can act as trapping centers for photo-generated electrons or holes and inhibit photo-generated charge complexes; (2) they act as active centers and promote the adsorption and activation of reactant molecules; (3) they adjust the band gap energy of catalysts and enhance the light absorption capacity.

**Figure 4:**
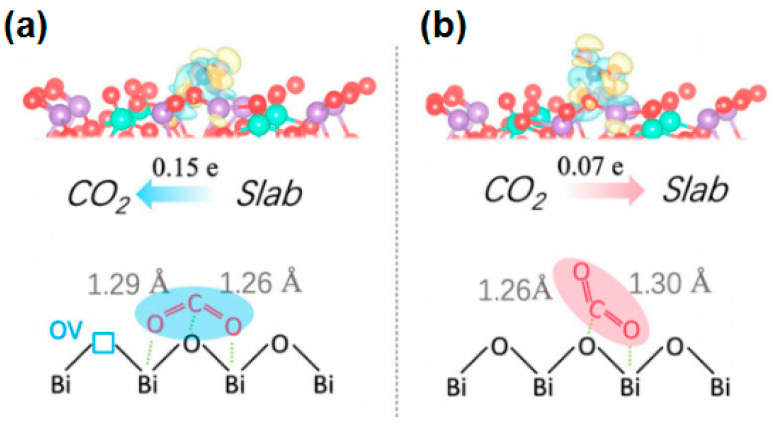
(**a**) Absorption of B1−CO_2_ on BMO OVs and (**b**) B2−CO_2_ on BMO [54]. Copyright 2019, Elsevier.

Researchers assembled ultra-thin HNb_3_O_8_ nanosheets and BiOBr nanosheets to prepare BiOBr/HNb_3_O_8_ Z-scheme heterojunctions with abundant oxygen vacancies [53]. The photocatalytic CO_2_ reduction performance of the photocatalyst was greatly improved, which was attributed to various synergies: (1) the 2D–3D structure produced a more active site; (2) abundant oxygen vacancies enhanced the light absorption ability; (3) the combination of Z-scheme heterostructures and oxygen vacancies promoted the separation and transfer of photogenerated charge carriers. The best BiOBr VO/HNB_3_O_8_ NS (50%-BiOBr VO/HNB_3_O_8_ NS) photocatalyst showed a CO yield of up to 164.61 μmol g^−1^ and a high selectivity of 98.7%. In addition, after five cycles, the catalyst exhibited excellent stability, indicating that the number of induced oxygen vacancies remained relatively balanced during the photocatalytic reaction due to the inhibitory effect of the Z-scheme heterostructure on the oxygen vacancy reduction process. Yang et al. synthesized Bi_2_MoO_6_ (BMO) with exposed {001} crystal planes by a simple solvothermal method [54]. Compared with Bi_2_MoO_6_, the yields of CO and CH_4_ on Bi_2_MoO_6_ containing oxygen vacancies (BMO OVs) reached 0.27 and 2.01 μmol g^−1^ h^−1^, respectively. Additionally, the corresponding CH_4_ selectivity was up to 96.7%. On this basis, the team explored the CO_2_ adsorption mode and reaction process, and for the first time revealed the hidden mechanism of CO_2_ selective conversion on oxygen vacancies. The calculation result of charge density difference showed that 0.15 electrons were returned from the slab to B1−CO_2_ after CO_2_ adsorption on the surface of BMO OVs, which depleted the local electrons of the two Bi atoms around the oxygen vacancy, and more electrons accumulated on B1−CO_2_ (Figure 4). In contrast, 0.07 electrons are transferred from CO_2_ to the slab in BMO. In addition, the C−O bond lengths of CO_2_ adsorbed on BMO OVs and BMO increase to 1.26 Å or 1.29 Å and 1.26 Å or 1.30 Å, respectively. In an overall analysis, the OVs on the surface of Bi_2_MoO_6_ {001} are more favorable to the chemisorption of CO_2_.

Vacancies on the catalyst surface are rich in localized electrons, which can transfer electrons to the adsorbed gas molecules on the surface and form coordination bonds between nearby metal atoms and reactants, activating the reactants and driving the photocatalytic reaction to proceed. Although the vacancy design can improve the CO_2_ reduction activity of catalysts, the long-term efficient stabilization of vacancy defects remains a challenge.

### 3.2. Morphological Control

**Figure 5:**
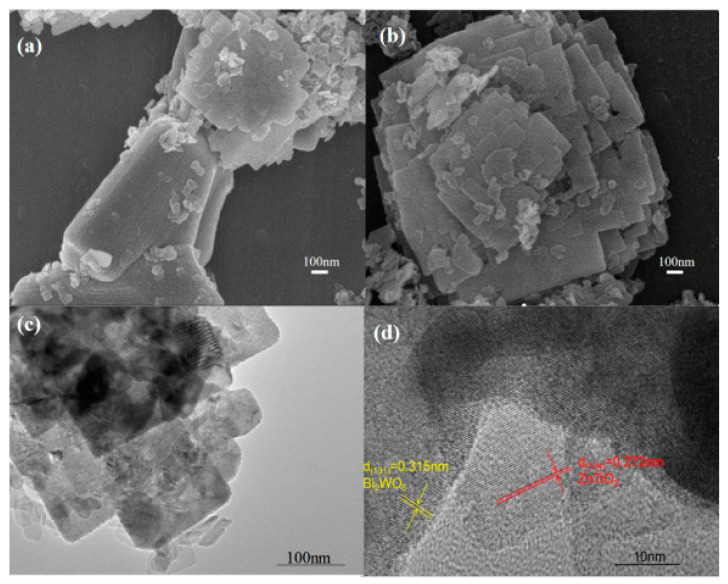
(**a**,**b**) SEM imagesZnTiO_3_/Bi_2_WO_6_ heterojunction sample, (**c**) TEM image and (**d**) HRTEM image of ZnTiO_3_/Bi_2_WO_6_ heterojunction sample [58].

There is a constitutive relationship between nanomaterials’ size, morphological structure, and properties. Particle size affects the band gap energy, light absorption capacity, and the material’s average free range of photo-generated charges. When the particle size of the material is smaller than the thickness of its space charge layer, the space charge layer is negligible, and the photo-generated charge migrates from the bulk phase to the material surface through simple diffusion and participates in the surface redox reaction. Photo-generated charge transfer and separation efficiency is improved by shortening the charge migration distance [55]. On the other hand, the smaller particle size with a high specific surface area and excellent adsorption properties can promote the interaction between catalysts and reactants while providing abundant active sites and a wide light absorption area for the redox reaction, enhancing photocatalytic performance [56]. The researchers prepared AgCl/δ-Bi_2_O_3_ nanosheets with a thickness of about 2.7 nm by the hydrothermal precipitation method [57]. The structural properties of the catalysts, including morphology, crystallinity, optical properties, and energy band structure, were analyzed by SEM, TEM, and AFM tests. The characterization results show that the ultrathin two-dimensional nanomaterials have a larger specific surface area and two-dimensional anisotropy, exposing more surface unsaturated paired atoms, increasing the active surface centers, and shortening the charge migration distance to achieve effective separation of electron-hole pairs. At the same time, surface atoms tend to escape from the lattice during the size reduction of nanomaterials, inducing the generation of surface defects. Jiang et al. [58] prepared S-Scheme 2D/2D heterojunction ZnTiO_3_ nanosheets/Bi_2_WO_6_ nanosheets as catalysts. The catalyst structures are shown in Figure 5.

Wu et al. [59] prepared hierarchical Z-scheme BiVO_4_/hm-C_4_N_3_ structures. The semiconductor material BiVO_4_ has a large specific surface area, which is favorable for trapping and absorbing incident photons. Due to the darker appearance of hm-C_4_N_3_, the BiVO_4_/hm-C_4_N_3_ composite exhibits significantly higher light absorption in the UV and visible regions. Z-scheme structure promotes the separation of *e*^−^-*h*^+^ and enhances the reduction ability of hm-C_4_N_3_. BiVO_4_/hm-C_4_N_3_ composites achieve photocatalytic CO_2_ reduction to CO (48.0 μmol·g^−1^h^−1^) with a selectivity of more than 97%. Lu et al. [60] prepared 2D/2D g-C_3_N_4_/BiVO_4_ Z-scheme catalysts. Due to the 2D–2D structure of g-C_3_N_4_ and BiVO_4_ nanosheets, without obvious boundaries, thus providing abundant active sites with effectively enhanced interfacial charge migration. It was shown that the prepared catalysts achieved CO and CH_4_ conversions of 5.19 μmol·g^−1^h^−1^ and 4.57 μmol·g^−1^h^−1^, respectively.

### 3.3. Heterojunction Construction

Heterostructure refers to the interfacial region formed by the contact of two different semiconductors. The construction of heterojunction enhances the light absorption ability of catalysts, improves the quantum efficiency by the window effect, and also promotes the separation of photo-generated electrons and holes by the interfacial effect. The structure is characterized by the fact that [61,62] (1) the surfaces of both components are exposed, which gives both particles the possibility to interact with the environment; (2) both materials form nanoparticles independently, making them more functional and have dual component properties. The heterostructure is a compounding of electrons from the more positive conduction band position and holes from the more negative valence band position of two semiconductors at the heterojunction interface, respectively. The remaining photo-generated electrons in the more negative CB and the remaining photo-generated holes in the more positive VB are simultaneously retained, and they have excellent reduction and oxidation abilities. Semiconductor photocatalysts with more negative CB positions can be considered excellent reduced photocatalysts, while those with more positive VB positions can be considered better oxidation photocatalysts. The composite heterojunction of reduced and oxidized photocatalysts could take full advantage of the high reduction and oxidation ability, thus significantly enhancing the photocatalytic performance.

**Figure 6:**
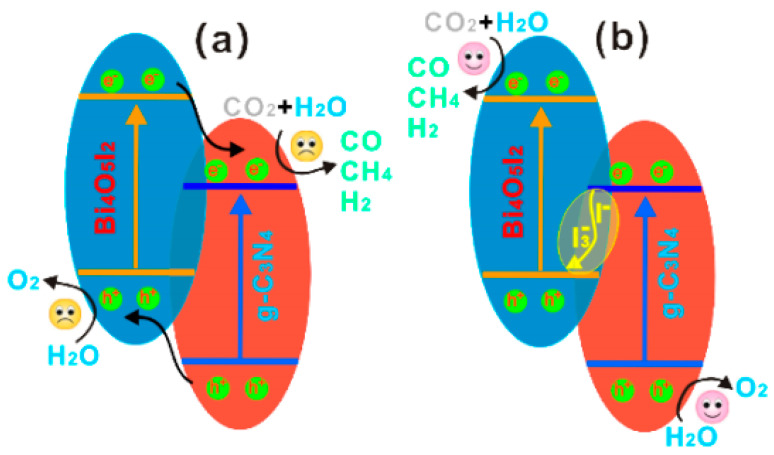
Photocatalytic enhancement mechanism for reducing CO_2_: (**a**) charge transfer mechanism, and (**b**) Z-scheme mechanism [63]. Copyright 2016, Elsevier.

Z-scheme heterojunction can achieve spatial separation of electrons and holes on different semiconductor materials, with advantages such as wide spectral response, high charge separation efficiency, strong redox ability, and high stability. Z-scheme heterojunction has broad prospects in the application of photocatalytic CO_2_ reduction. The common Z-scheme heterojunction consists of two band matching semiconductors (I and Ⅱ) and redox medium. The CB and VB positions of semiconductor I are higher than those of semiconductor Ⅱ, and the redox medium serves as the interface charge transfer channel between the two. Under light excitation conditions, both semiconductors are excited to generate electrons and holes, which remain in their CB and VB respectively. The excited electrons in the CB of semiconductor I have strong reduction ability and can catalyze the reduction of CO_2_; the photogenerated holes in the VB of semiconductor Ⅱ have strong oxidation ability and can catalyze the oxidation of H_2_O. The electrons in the CB of semiconductor Ⅱ and the holes in the VB of semiconductor I will be transferred through the electronic medium and annihilated. Therefore, the Z-scheme heterojunction effectively inhibits the recombination of electron hole pairs and significantly improves photocatalytic performance. Bai et al. [63] reported a g-C_3_N_4_/Bi_4_O_5_I_2_ heterojunction photocatalyst prepared by precursor hydrolysis and applied to the visible light reduction of CO_2_ for fuel production. Oxygen-active species quantification experiments, confirmed that the heterojunction enhances the efficiency of photocatalytic CO_2_ reduction by generating an I^−^/I^3−^ redox mediator with a direct Z-scheme structure. Combined with the test results of ·OH, it can be inferred that the H_2_O oxidation position of g-C_3_N_4_/Bi_4_O_5_I_2_ should be the VB of g-C_3_N_4_ rather than the VB of Bi_4_O_5_I_2_. So the charge transfer mechanism in Figure 6a does not work. This finding further confirms the previous inference about the direct Z-scheme structure of the bismuth iodide oxygen composite (Figure 6b). Under visible light irradiation, the photogenerated electrons in the photocatalyst are transferred from the CB of g-C_3_N_4_ to the VB of Bi_4_O_5_I_2_ through the I_3_^−^/I^−^ redox medium, promoting the separation of photogenerated electrons and holes while retaining strong redox ability. Therefore, g-C_3_N_4_/Bi_4_O_5_I_2_ exhibits high photocatalytic reduction performance, with a yield of 45.6 μmol h^−1^ g^−1^ for CO_2_ conversion to CO.

**Figure 7:**
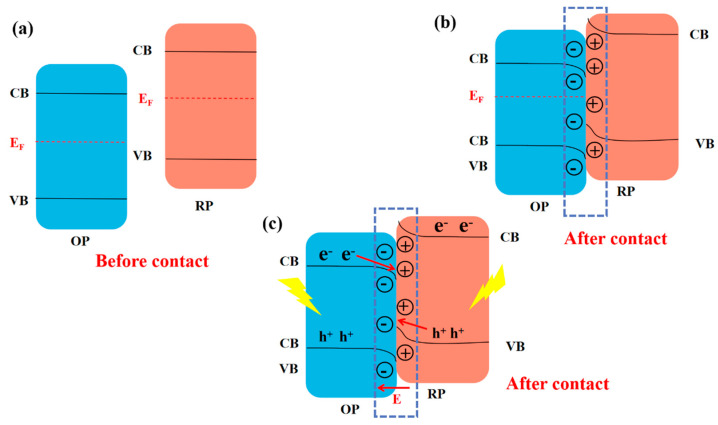
Charge transfer processes in the S-scheme structure [33]. Copyright 2022, Elsevier.

Guo et al. [64] constructed in situ S-scheme (Figure 7a–c) BiOBr/Bi_2_WO_6_ heterojunction photocatalysts with tight interfacial contacts using a one-step hydrothermal method. The unique nanoflower morphology was obtained by tuning the synthesis conditions. The material was further calcined under a nitrogen atmosphere to introduce surface oxygen vacancies. Without any sacrificial agent and co-catalyst, the heterojunction material exhibited excellent photocatalytic CO_2_ reduction with a product CO production rate of 55.17 μmol·g^−1^ h^−1^, higher than most reported photocatalysts. The reason is that the construction of S-scheme heterojunction greatly facilitates photo-generated charges’ separation and transfer efficiency while having an efficient redox capability. The nanoflower morphology substantially enhances the CO_2_ adsorption capacity of the photocatalyst due to its large specific surface area. The efficiency of photocatalytic CO_2_ reduction is greatly improved. Miao et al. [65] prepared a catalyst with BiOBr/Bi_2_S_3_ S-scheme structure by the hydrothermal method. Since the Fermi energy level of Bi_2_S_3_ is larger than BiOBr, and the power function is smaller than BiOBr, when the electrons of Bi_2_S_3_ come into contact with BiOBr, they could transfer electrons to BiOBr and form an internal electric field. Under light irradiation, the photo-generated holes on Bi_2_S_3_ VB are recombined with the photo-generated electrons on BiOBr CB driven by the internal electric field, and the photo-generated electrons on BiOBr CB are used for the CO_2_ reduction reaction. The CO and CH_4_ yields of the best photocatalyst were as high as 100.8 and 8.5 μmol·g^−1^h^−1^, respectively.

### 3.4. Co-Catalyst Loading

Photocatalytic CO_2_ conversion could be broadly divided into three processes [66,67,68]: (i) absorption of light by the catalyst to generate electron–hole pairs, (ii) separation and transfer of electron–hole pairs to the catalyst surface, and (iii) surface reactions of H_2_O oxidation and CO_2_ reduction. Adding a co-catalyst could enhance the process (iii) and improve the photocatalytic activity. Introducing a co-catalyst into the host lattice of a semiconductor induces defective states in the electronic and chemical structure, which in turn affects the overall performance of the catalyst. In photocatalytic CO_2_ reduction, the critical roles of doping sites are to act as active centers for CO_2_ adsorption activation and to promote photo-generated charge separation. The co-catalyst has the following three critical roles: (1) promoting the separation of photoexcited electron–hole pairs, (2) inhibiting side reactions, and (3) improving the selectivity of the target product. Jiang et al. [69] loaded Pt nanoparticles as co-catalysts onto the surface of the prepared CsPbBr_3_/Bi_2_WO_6_ catalyst. The ultrathin nanosheet structure gave the catalyst a short charge transfer distance, thus facilitating charge separation. In addition, Bi_2_WO_6_ NSs provide additional electron transfer channels, which effectively inhibit charge recombination. It was shown that the selectivity of CO and CH_4_ accounted for 11.4% and 84.3%, respectively. The activity of the loaded catalysts was increased by 2.4 times compared with those prepared without the Pt nanoparticle co-catalysts.

In addition to the noble metal Pt, other noble metals (such as Au and Ag) could be loaded to enhance the photocatalytic CO_2_ reduction activity. Precious metal co-catalysts have a selective effect on the reactant intermediates, which in turn affects the selectivity of the products. Yoshino et al. [70] loaded Cu, Ag, Ru, Au, and Pt onto BiVO_4_ to photocatalytically reduce CO_2_. The results showed that the catalysts prepared by loading Ag or Au exhibited excellent photocatalytic CO_2_ reduction performance compared to Ru, Cu, and Pt. The reason is that Ag or Au has excellent photoelectric activity.

Therefore, the activity, selectivity, and stability of the co-catalysts depend primarily on the preparation and dispersion methods, as these methods directly affect the physicochemical properties (e.g., chemical composition, size, morphology) of the co-catalysts.

## 4. Multiple Bi-Based Photocatalysts for CO_2_ Reduction

Over the past 50 years, more than 150 semiconductors, including metal oxides, metal sulfides, carbon based materials, and MOF, have made significant progress in environmental remediation. Among them, Bi-based semiconductors have attracted much attention due to their unique layered structure, tunable electronic and visible light response performance, and great potential in energy conversion and environmental remediation applications.

### 4.1. Binary Bi-Based Semiconductor

Compared with other Bi-based materials, Bi_2_O_3_ has a suitable band gap (2.1–2.8 eV) [71]. Bi_2_O_3_ has α-, β-, γ-, δ-, ω-, ε- six polymorphic forms. Among them, β-Bi_2_O_3_ exhibits relatively excellent photocatalytic activity, which is attributed to its narrow bandgap and strong light absorption ability. However, Bi_2_O_3_ faces the problem of easy recombination of photo generated carriers when used as a photocatalyst, so it is often coupled with other semiconductors to construct heterojunctions for use. Liu et al. prepared Bi_2_Al_4_O_7_/β-Bi_2_O_3_ heterostructures through the one-step in situ auto-combustion method and successfully introduced oxygen vacancy in the synthesis process [72]. Bi_2_Al_4_O_7_/β-Bi_2_O_3_ showed high photocatalytic capacity in the photocatalytic reduction of CO_2_ to CO under the synergistic action of heterojunction photogenerated carrier separation and oxygen vacancy activation of CO_2_. Compared with the original β-Bi_2_O_3_, the CO yield of 0.14BAB catalyst is 13.2 μmol/g, which is an 8-fold increase. Compared with Bi_2_O_3_, Bi_2_S_3_ has a narrower bandgap (~1.3 eV) and higher absorption coefficient. Therefore, Bi_2_S_3_ often acts as a visible light absorber in the composition of photocatalysts. For example, Bi_2_S_3_ combines with TiO_2_, g-C_3_N_4_, and ZnIn_2_S_4_ to photocatalytic reduce CO_2_ to value-added chemicals such as methanol [73].

### 4.2. Ternary Bi-Based Semiconductors

Bismuth halide oxide (BiOX, X = Cl, Br, I) has been extensively studied in photocatalysis due to its excellent photocatalytic properties, environmental friendliness, and low price. The layered structure of BiOX provides enough space to polarize the associated atoms and orbitals, which excites the formation of an internal electric field between [Bi_2_O_2_] and halogen. The internal electric field can accelerate the separation and migration of photoexcited electron–hole pairs, and the photocatalytic activity of BiOX is significantly improved. Kong et al. [74] prepared BiOBr nanosheets containing oxygen vacancies to enhance the catalyst activity by ethylene glycol-assisted solvothermal method. The total CH_4_ yields were obtained at 4.86 µmol·g^−1^ and 9.58 µmol·g^−1^ for visible light irradiation and simulated sunlight irradiation, respectively. In contrast, the total CH_4_ yields were only 1.58 and 2.99 µmol·g^−1^ for BiOBr without oxygen vacancies, respectively. The surface oxygen vacancies adsorb and activate CO_2_, which reduces the energy barrier for charge transfer. It also has a strong ability to trap photo-generated electrons, which reduces the probability of electron–hole complexation. Therefore, in the preparation of bismuth halide oxide catalysts, oxygen vacancies could be introduced on the catalyst surface to improve the activity of the catalyst.

### 4.3. Quaternary Bi-Based Semiconductors

Bi_2_WO_6_ is one of the simplest Aurivillius oxides, with a perovskite-like [WO_4_]^2−^ layer sandwiched between bismuth oxide [Bi_2_O_2_]^2+^ layers [75]. The CB of Bi_2_WO_6_ is composed of W 5d orbitals, while its VB is composed of a hybrid of O 2p and Bi 6s orbitals, resulting in a narrower bandgap and a visible light response ability. Bi_2_WO_6_ has very interesting physical and chemical properties, such as ferroelectricity, catalytic activity and nonlinear dielectric magnetic susceptibility. In recent years, Bi_2_WO_6_ has attracted widespread attention as a potential candidate for visible light-induced photocatalysts and photoelectrochemical (PEC) CO_2_ reduction. For example, the Bi_2_WO_6_ layer was prepared by Liang et al. [76] using oleate reacted with Bi to form a layered Bi-oleic acid complex and then adding sodium tungstate, which has excellent photocatalytic activity. The CO_2_ reduction activity was increased by nearly 130 times. Wang’s team prepared Bi_2_WO_6_/BiOCl heterojunction (BW-X) in situ on F-SnO_2_ transparent conductive glass by hydrothermal method and applied it to PEC CO_2_ reduction [77]. The BCW-X heterojunction had a excellent 2D layered/3D flower structure, and the exposed crystal surface of BiOCl had changed from the original (101) to (112) in the heterojunction, improving the separation efficiency of photogenerated electron–hole. Under simulated sunlight exposure, the BCW-X electrode in the BCW-X|KHCO_3_|BiVO_4_ PEC cell showed excellent ability to convert water and CO_2_ molecules into hydrocarbons at −1.0 V. Ethanol was produced at a rate of 11.4 μM h^−^^1^ cm^−^^2^ (600 μmol h^−^^1^ g^−^^1^) with a selectivity of 80.0%. The apparent quantum efficiency was as high as 0.63%, about 3 times that of the composite BiOCl-Bi_2_WO_6_ photocathode.

BiVO_4_ is an N-type semiconductor material with a narrow band gap of ~2.4 eV. BiVO_4_ has low toxicity and excellent visible-light responsive activity, exhibiting high activity for solar-driven water splitting and organic decomposition, which has attracted widespread interest in the scientific community. More attractive is that BiVO_4_ has an appropriate band edge, which makes it suitable for water oxidation coupled with CO_2_ reduction [78]. Li et al. [79] prepared 2D/2D BiVO_4_/Ti_3_C_2_T_x_ catalysts to verify the role of the surface contact interface on photocatalytic CO_2_ reduction. Photoexcitation of BiVO_4_ effectively transfers electrons to Ti_3_C_2_T_x_ through the formation of Schottky barriers. TEM observed electrostatic positive and negative charges on BiVO_4_ nanosheets and Ti_3_C_2_T_x_ flakes, and the prepared catalysts showed a robust surface contact interface. In addition, the transband alignment of BiVO_4_ with water redox levels makes it a valuable visible active photoanode for CO_2_ reduction systems [80]. However, BiVO_4_ still needs to overcome serious internal obstacles, such as poor electron transport and slow oxidation kinetics, to meet practical application requirements. For efficient CO_2_ reduction performance, a suitable photoanode with a low initial potential value is required to minimize the use of an external power source and drive the process primarily through solar energy. Therefore, BiVO_4_-based photoelectrodes with reduced initial potential and improved photocurrent are expected to develop superior CO_2_ reduction systems.

A number of specialized words appear in the paper, and the following table shows the abbreviations of the professional terms (Table 2). In addition, recent reports on the performance efficiencies associated with Bi-based photocatalysts are summarized in Table 3.

## 5. Conclusions

Photocatalytic CO_2_ reduction is a promising way to utilize CO_2_. It can use inexhaustible solar energy to convert CO_2_ into high-value-added chemicals. Impressive results have been achieved. The selection of highly active, selective, and stable materials is necessary to improve CO_2_ reduction activity. Bi-based photocatalysts have great potential in reducing CO_2_ to fuel under visible light due to unique electronic structure, crystal structure, and physical and chemical properties. In this paper, the recent progress of modification of Bi-based photocatalyts to improve their photocatalytic properties is reviewed. Many optimization strategies were discussed in detail to achieve strong photocatalytic performance, such as heterojunction, introduction of vacancies, morphology adjustment, and co-catalyst loading. Although significant improvements in photocatalytic efficiency have been achieved, there are still many opportunities and challenges for Bi-based photocatalysts.

1. There is currently no unified explanation about the reaction mechanism of Bi-based photocatalyst. The active species of catalyst in the reaction is controversial. For example, Bi is easily oxidized to form a thin layer of bismuth oxide in the surrounding environment. The presence of bismuth oxide on the catalyst surface and its influence on the photocatalytic process remain unclear. In the future, more attention should be paid to key issues such as the active site of reactants, charge transfer dynamics, and molecular orbital. Some advanced characterization techniques with high spatial, temporal, and spectral resolution, such as in situ XPS, X-ray absorption near-side structures XANES, and EXAFS, need to be introduced into the research to analyze the reaction process from the microscopic level. In addition, in situ analysis technology can be used to monitor the changes of catalyst and product in real time during the reaction process, thereby inferring the reaction pathway.

2. Due to the multiple reaction steps involved in CO_2_ reduction reaction, various products are formed, leading to selectivity issues. From recent research trends, it is not difficult to find that the main products of Bi-based materials are concentrated in C_1_ derivatives, such as CO, methane, formic acid, methanol. C_2+_ derivatives with higher added value, such as ethylene, ethanol, ethane, propanol, and acetone, are rarely reported due to their more difficult generation. Therefore, an effective Bi-based photocatalyst should be designed to selectively produce C_2+_ products.

3. The stability of Bi-based materials is a fundamental factor determining the lifespan and performance of photocatalysts. The prerequisite for developing efficient and stable photocatalysts is to suppress the poisoning and deactivation of Bi-based materials. Usually, researchers conduct multiple cyclic experiments in performance testing to evaluate the stability of the catalyst. However, the testing time is often limited to the range of a few hours to dozens of hours, far from meeting the requirements of industrial applications. It is insufficient to rely solely on the final reaction results in loop testing. The changes in the surface structure of Bi-based materials during the reaction process also need to be monitored and studied. In addition, the impact of photo corrosion on Bi-based materials in photocatalytic reactions also needs to be further studied.

4. The ultimate goal of photocatalytic research is to achieve industrial applications, and the preparation of catalysts in a sustainable manner is an inevitable problem to be faced and solved. At present, the quantity of Bi-based materials prepared is still relatively small, and the yield of photocatalysts prepared by existing methods is often less than a few grams. The method of large-scale production of Bi-based materials with excellent performance is not yet mature. Therefore, in order to meet the production requirements, a synthesis method with low cost and simple preparation process should be developed to prepare high efficiency and high stability of Bi-based photocatalyst, making it possible to realize practical application in the field of photocatalysis and energy sustainability.

## Figures and Tables

**Table 1:** Photocatalytic CO_2_ reduction products and corresponding electrode potentials.

Reaction	Reduction Potential (vs. NHE at pH = 7)
CO_2_ + 2H^+^ + 2*e^−^* → HCOOH	−0.61
CO_2_ + 2H^+^ + 2*e^−^* → CO + H_2_O	−0.53
CO_2_ + 4H^+^ + 4*e^−^* → HCHO + H_2_O	−0.48
CO_2_ + 4H^+^ + 4*e^−^* → C + 2H_2_O	−0.20
CO_2_ + 6H^+^ + 6*e^−^* → CH_3_OH + H_2_O	−0.38
CO_2_ + 8H^+^ + 8*e^−^* → CH_4_ + 2H_2_O	−0.24
2CO_2_ + 2H^+^ + 2*e^−^* → C_2_H_4_ + 4H_2_O	−0.34
CO_2_ + 2H^+^ + 2*e^−^* → CH_3_OH + 3H_2_O	−0.33
2CO_2_ + 14H^+^ + 14*e^−^* → C_2_H_6_ + 4H_2_O	−0.27
2H^+^ + 2*e^−^* → H_2_	−0.42

**Table 2:** Abbreviations of professional terms.

Names	Abbreviations
Valence band	VB
Conduction band	CB
Lowest unoccupied molecular orbital	LUMO
Highest occupied molecular orbital	HOMO

**Table 3:** Summary of photocatalytic CO_2_ reduction performance with Bi-based materials.

Catalysts	Preparation Method	Reaction Condition		Major Products	Production Rate /µmol gcat^−1^ h^−1^	Ref.
		Light Source	Solution			
ultrathin Bi_4_O_5_Br_2_	precursor method	300 W high-pressure xenon lamp	CO_2_/H_2_O vapor	CO	31.57 μmol g^−1^ h^−1^	[81]
Ag-Bi/BiVO_4_	galvanic replacement reaction	300 W Xe-illuminator with a light cutoff filter (λ > 420 nm)	0.5 mL H_2_O deionized water	CO	~5.19 μmol g^−1^ h^−1^	[82]
a-BiOCl	liquid exfoliation	300 W Xenon arc lamp with a filter (AM 1.5 G)	50 mg of catalyst and 100 mL of Milli-Q water	CO	8.99 µmol g^−1^ h^−1^	[83]
Bi/Bi_2_SiO_5_	one-step hydrothermal strategy	50 mL of deionized water	300 W Xe-lamp	CO	33.02 µmol g^−1^ h^−1^	[84]
Cu-Bi/BiVO_4_	solvothermal method	1 mL distill water	Xe lamp with a light intensity of 160 mW·cm^2^ and a wavelength range of 420~780 nm	CO	11.15 μmol h^−1^ g^−1^	[85]
Bi/Bi_2_SiO_5_	OH^−^-assisted hydrothermal controllable route	50 mL of deionized water	300 W Xe-lamp (200–2500 nm)	CO	62.70 μmol h^−1^ g^−1^	[86]
Bi/CsPbBr_3_	in-situ growth method	0.5 mL of deionized water	300 W Xenon lamp	CO	19.1 µmol g^−1^ h^−1^	[87]
Bi/AgBiS_2_/P25	one-step solvothermal treatment	4 mL of deionized water	300 W Xenon lamp	CH_4_ (CO)	4.31 µmol g^−1^ h^−1^ (6.37 µmol g^−1^ h^−1^)	[88]
Bi_2_O_2_CO_3_/Bi/NiAl-LDH	hydrothermal method	100 mL deionized water	300 W Xenon lamp with a cut−800 nm filter	CH_4_	56.64 μmol g_cat_^−1^	[89]
P/Bi-BiOBr	in-situ bismuth deposition and phosphorus modification	2.5 mL H_2_O	300 W xenon lamp with 400 nm filter	CH_4_	0.62 µmol g^−1^ h^−1^	[90]
Bi_2_O_2_S	hydrothermal method	1.2 g of Na_2_CO_3_ and 2 mL of H_2_SO_4_ (1:1 vol.)	300 W of Xe lamp with a 420-nm filter	CH_4_	43.87 µmol g^−1^ h^−1^	[91]
Bi_2_MoO_6_	sonication-assisted chemical reduction	20 mg of photocatalyst and 5 mL water	300 W Xenon lamp	CH_4_ (CO)	12.4 µmol g^−1^ h^−1^ (61.5 µmol g^−1^ h^−1^)	[92]
In_2_O_3_/BiOI	solvothermal methods	TEOA as a sacrificial agen	300 W Xe lamp with a cut-off filter (λ ≥ 420 nm)	CH_4_ (CO)	5.69 µmol g^−1^ h^−1^ (11.98 µmol g^−1^ h^−1^)	[93]
TiO_2_@BiOCl	chemical impregnation and calcination	5 μL acetonitrile and 1 mL H_2_O	300 W high pressure xenon lamp	CH_4_	168.5 µmol g^−1^ h^−1^	[94]
surface iodinated Bi_2_O_2_S	hydrothermal reaction	1.2 g of Na_2_CO_3_ and 2 mL H_2_SO_4_ (1:1 vol)	Xe lamp (300 W) with a 420 nm cutoff filter	CH_4_	53.35 µmol g^−1^ h^−1^	[95]
Ti_3_C_2_/Bi_2_WO_6_	etching and ultrasonic exfoliation	0.084 g NaHCO_3_ and 0.3 mL H_2_SO_4_ (2 mol L^−1^)	Xe lamp (300 W)	CH_4_	1.78 µmol g^−1^ h^−1^	[96]
Bi@Bi_2_MoO_6_	solvothermal approach	Xe lamp (λ ≥ 400)	30 mL uniform solution containing 50 mg of photocatalyst and 0.42 g NaHCO_3_	C_2_H_5_OH	17.93 µmol g^−1^ h^−1^	[97]

## Data Availability

Not applicable.

## Sample Availability

Samples of the compounds are available from the authors.

## References

[1] Habisreutinger S.N., Schmidt-Mende L., Stolarczyk J.K. (2013). Photocatalytic Reduction of CO_2_ on TiO_2_ and Other Semiconductors. Angew. Chem. Int. Ed..

[2] Shinde G.Y., Mote A.S., Gawande M.B. (2022). Recent Advances of Photocatalytic Hydrogenation of CO_2_ to Methanol. Catalysts.

[3] Fujishima A., Honda L. (1972). Electrochemical photolysis of water at a semiconductor electrode. Nature.

[4] Li X., Fang Y., Wang J., Fang H., Xi S., Zhao X., Xu D., Xu H., Yu W., Hai X. (2021). Ordered clustering of single atomic Te vacancies in atomically thin PtTe_2_ promotes hydrogen evolution catalysis. Nat. Commun..

[5] Jiang Z.Y., Sun W., Miao W.K. (2019). Living Atomically Dispersed Cu Ultrathin TiO_2_ Nanosheet CO_2_ Reduction Photocatalyst. Adv. Sci..

[6] Kumar A., Sharma G., Naushad M., Ahamad T., Veses R.C., Stadler F.J. (2019). Highly visible active Ag_2_CrO_4_/Ag/BiFeO_3_@RGO nano-junction for photoreduction of CO_2_ and photocatalytic removal of ciprofloxacin and bromate ions: The triggering effect of Ag and RGO. Chem. Eng. J..

[7] Zhao Q., Sun J., Li S.C., Huang C.P., Yao W.F., Chen W., Zeng T., Wu Q., Xu Q.J. (2018). Single nickel atoms anchored on nitrogen-doped graphene as a highly active cocatalyst for photocatalytic H_2_ evolution. ACS Catal..

[8] Fu J., Xu Q., Low J., Jiang C., Yu J. (2019). Ultrathin 2D/2D WO_3_/g-C_3_N_4_ step-scheme H_2_-production photocatalyst. Appl. Catal B.

[9] Yu J.G., Low J.X., Xiao W., Zhou P., Jaroniec M. (2014). Enhanced photocatalytic CO_2_ reduction activity of anatase TiO_2_ by coexposed {001} and {101} facets. J. Am. Chem. Soc..

[10] Wang Z., Teramura K., Huang Z., Hosokawa S., Sakata Y., Tanaka T. (2016). Tuning the selectivity toward CO evolution in the photocatalytic conversion of CO_2_ with H_2_O through the modification of Ag-loaded Ga_2_O_3_ with a ZnGa_2_O_4_ layer. Catal. Sci. Technol..

[11] Yu J., Jin J., Cheng B., Jaroniec M.A. (2014). noble metal-free reduced graphene oxide-CdS nanorod composite for the enhanced visible-light photocatalytic reduction of CO_2_ to solar fuel. J. Mater. Chem. A.

[12] Yuan Y., Guo R.-T., Hong L.-F., Ji X.-Y., Li Z.-S., Lin Z.-D., Pan W.-G. (2021). Recent advances and perspectives of MoS_2_-based materials for photocatalytic dyes degradation: A review. Colloids Surf. A Physicochem. Eng. Asp..

[13] An X., Li K., Tang J. (2014). Inside Cover Picture: Cu_2_O/Reduced Graphene Oxide Composites for the Photocatalytic Conversion of CO_2_. ChemSusChem.

[14] Shown I., Hsu H.C., Chang Y.C., Lin C.H., Roy P.K., Ganguly A., Wang C.H., Chang J.K., Wu C.I., Chen L.C. (2014). Highly Efficient Visible Light Photocatalytic Reduction of CO_2_ to Hydrocarbon Fuels by Cu-Nanoparticle Decorated Graphene Oxide. Nano Lett..

[15] Zhou Y., Tian Z., Zhao Z., Liu Q., Kou J., Chen X., Gao J., Yan S.C., Zou Z.G. (2011). High-yield snthesis of ultrathin and uniform Bi_2_WO_6_ square nanoplates benefitting from photocatalytic reduction of CO_2_ into renewable hydrocarbon fuel under visible light. ACS Appl. Mater. Interfaces.

[16] Gondal M.A., Dastageer M.A., Oloore L.E., Baig U. (2017). Laser induced selective photo-catalytic reduction of CO_2_ into methanol using In_2_O_3_-WO_3_ nano-composite. J. Photochem. Photobiol. A.

[17] Mao J., Li K., Peng T. (2013). Recent advances in the photocatalytic CO_2_ reduction over semiconductors. Catal. Sci. Technol..

[18] Zhu C.Q., Wang Q.N., Wu C. (2022). Rapid and scalable synthesis of bismuth dendrites on copper mesh as a high-performance cathode for electroreduction of CO_2_ to formate. J. CO_2_ Util..

[19] Wu D., Chen W.Y., Wang X.W., Fu X.Z., Luo J.L. (2020). Metal-support interaction enhanced electrochemical reduction of CO_2_ to formate between graphene and Bi nanoparticles. J. CO_2_ Util..

[20] Choi K.M., Kim D., Rungtaweevoranit B., Trickett C.A., Barmanbek J.T., Alshammari A.S., Yang P., Yaghi O.M. (2017). Plasmon-enhanced photocatalytic CO_2_ conversion within metal-organic frameworks under visible light. J. Am. Chem. Soc..

[21] Zhang Y., Wang L., Dong F., Chen Q., Jiang H., Xu M., Shi J. (2019). Non-additional carbon source one-step synthesis of Bi_2_O_2_CO_3_-based ternary composite for efficient Z-scheme photocatalysis. J. Colloid. Interf. Sci..

[22] Zhao Z.Y., Dai W.W. (2015). Electronic structure and optical properties of BiOI ultrathin films for photocatalytic water splitting. Inorg. Chem..

[23] Hong L.F., Guo R.T., Yuan Y., Ji X.Y., Lin Z.D., Li Z.S., Pan W.G. (2021). Recent progress of transition metal phosphides for photocatalytic hydrogen evolution. ChemSusChem.

[24] Marcì G., García-López E.I., Palmisano L. (2014). Photocatalytic CO_2_ reduction in gassolid regime in the presence of H_2_O by using GaP/TiO_2_ composite as photocatalyst under simulated solar ligh. Catal. Commun..

[25] Sun Z., Wang H., Wu Z., Wang L. (2018). G-C_3_N_4_ based composite photocatalysts for photocatalytic CO_2_ reduction. Catal. Today.

[26] Que M., Cai W., Chen J., Zhu L., Yang Y. (2021). Recent advances in g-C_3_N_4_ composites within four types of heterojunctions for photocatalytic CO_2_ reduction. Nanoscale.

[27] Wang X., Maeda K., Thomas A., Takanabe K., Xin G., Carlsson J.M., Domen K., Antonietti M. (2008). A metal-free polymeric photocatalyst for hydrogen production from water under visible light. Nat. Mater..

[28] Cao S., Yu J. (2014). G-C_3_N_4_-based photocatalysts for hydrogen generation. J. Phys. Chem. Lett..

[29] Wu J., Xie Y., Ling Y., Si J.C., Li X., Wang J.L., Ye H., Zhao J.S., Li S.Q., Zhao Q.D. (2020). One-step synthesis and Gd^3+^ decoration of BiOBr microspheres consisting of nanosheets toward improving photocatalytic reduction of CO_2_ into hydrocarbon fuel. Chem. Eng. J..

[30] Zhao Z.Y., Dai W.W. (2014). Structural, electronic, and optical properties of Eu-doped BiOX (X = F, Cl, Br, I): A DFT+U study. Inorg. Chem..

[31] Xie S., Ma W., Wu X., Zhang H., Zhang Q., Wang Y., Wang Y. (2021). Photocatalytic and Electrocatalytic Transformations of C1Molecules Involving C-C Coupling. Energy Environ. Sci..

[32] Ajmal S., Yang Y., Tahir M.A., Li K., Bacha A.U.R., Nabi I., Liu Y., Wang T., Zhang L. (2020). Boosting C_2_ Products in Electrochemical CO_2_ Reduction over Highly Dense Copper Nanoplates. Catal. Sci. Technol..

[33] Hu X., Guo R.T., Chen X., Bi Z.X., Wang J., Pan W.G. (2022). Bismuth-based Z-scheme structure for photocatalytic CO_2_ reduction: A review. J. Environ. Chem. Eng..

[34] Hirano K., Inoue K., Yatsu T. (1992). Photocatalysed reduction of CO_2_ in aqueous TiO_2_ suspension mixed with copper powder. J. Photochem. Photobiol. A.

[35] Adachi K., Ohta K., Mizuno T. (1994). Photocatalytic reduction of carbon dioxide to hydrocarbon using copper-loaded titanium dioxide. Sol. Energy.

[36] Wang Z.-Y., Chou H.-C., Wu J.C., Tsai D.P., Mul G. (2010). CO_2_ photoreduction using NiO/InTaO_4_ in optical-fiber reactor for renewable energy. Appl. Catal. A Gen..

[37] Li Y.Y., Fan J.S., Tan R.Q., Yao H.C., Peng Y., Liu Q.C., Li Z.J. (2020). Recent Advances in TiO_2_-Based Photocatalysts for Reduction of CO_2_ to Fuels. Nanomaterials.

[38] Song Y., Chen W., Wei W., Sun Y. (2020). Advances in Clean Fuel Ethanol Production from Electro-, Photo- and Photoelectro-Catalytic CO_2_ Reduction. Catalysts.

[39] Xie Q., He W., Liu S., Li C., Zhang J., Wong P.K. (2020). Bifunctional S-scheme g-C_3_N_4_/Bi/BiVO_4_ hybrid photocatalysts toward artificial carbon cycling. Chin. J. Catal..

[40] Liu L., Dai K., Zhang J., Li L. (2021). Plasmonic Bi-enhanced ammoniated alpha-MnS/Bi_2_MoO_6_ S-scheme heterostructure for visible-light-driven CO_2_ reduction. J. Colloid Interface Sci..

[41] Wang Q.T., Fang Z.X., Zhang W., Zhang D. (2020). High-Efciency g-C_3_N_4_ Based Photocatalysts for CO_2_ Reduction: Modifcation Methods. Adv. Fiber Mater..

[42] Li X., Li W., Zhuang Z., Zhong Y., Li Q., Wang L. (2012). Photocatalytic Reduction of Carbon Dioxide to Methane over SiO_2_-Pillared HNb_3_O_8_. J. Phys. Chem. C.

[43] Li K., Peng B., Peng T. (2016). Preparation of AgIn_5_S_8_/TiO_2_ Heterojunction Nanocomposite and Its Enhanced Photocatalytic H_2_ Production Property under Visible Light. ACS Catal..

[44] Yu Y.Y., Dong X.A., Chen P., Geng Q., Wang H., Li J.Y., Zhou Y., Dong F. (2021). Synergistic Effect of Cu Single Atoms and Au-Cu Alloy Nanoparticles on TiO_2_ for Efficient CO_2_ Photoreduction. ACS Nano.

[45] Inoue H., Moriwaki H., Maeda K., Yoneyama H. (1995). Photoreduction of carbon dioxide using chalcogenide semiconductor microcrystals. J. Photochem. Photobiol. A.

[46] Kim C., Cho K.M., Al-Saggaf A., Gereige I., Jung H.-T. (2018). Z-scheme photocatalytic CO_2_ conversion on three-dimensional BiVO_4_/Carbon-coated Cu_2_O nanowire arrays under visible light. ACS Catal..

[47] Jo W.-K., Kumar S., Eslava S., Tonda S. (2018). Construction of Bi_2_WO_6_/RGO/g-C_3_N_4_ 2D/2D/2D hybrid Z-scheme heterojunctions with large interfacial contact area for efficient charge separation and high-performance photoreduction of CO_2_ and H_2_O into solar fuels. Appl. Catal. B.

[48] Hu X., Hu J., Peng Q., Ma X., Dong S., Wang H. (2020). Construction of 2D all-solid-state Z-scheme g-C_3_N_4_/BiOI/RGO hybrid structure immobilized on Ni foam for CO_2_ reduction and pollutant degradation. Mater. Res. Bull..

[49] Li X., Wei D., Ye L., Li Z. (2019). Fabrication of Cu_2_O-RGO/BiVO_4_ nanocomposite for simultaneous photocatalytic CO_2_ reduction and benzyl alcohol oxidation under visible light. Inorg. Chem. Commun..

[50] Dong F., Li Q., Sun Y., Ho W.-K. (2014). Noble metal-like behavior of plasmonic Bi particles as a cocatalyst deposited on (BiO)_2_CO_3_ microspheres for efficient visible light photocatalysis. ACS Catal..

[51] Zhao Y., Chen G., Bian T., Zhou C., Waterhouse G.I., Wu L.Z., Tung C.H., Smith L.J., O’Hare D., Zhang T. (2015). Defect-rich ultrathin ZnAl-layered double hydroxide nanosheets for efficient photoreduction of CO_2_ to CO with water. Adv. Mater..

[52] Wang S., Pan L., Song J.J., Mi W., Zou J.J., Wang L., Zhang X. (2015). Titanium-defected undoped anatase TiO_2_ with p-type conductivity, room-temperature ferromagnetism, and remarkable photocatalytic performance. J. Am. Chem. Soc..

[53] Zhou C., Shi X., Li D., Song Q., Zhou Y., Jiang D., Shi W. (2021). Oxygen vacancy engineering of BiOBr/HNb_3_O_8_ Z-scheme hybrid photocatalyst for boosting photocatalytic conversion of CO_2_. J. Colloid Interface Sci..

[54] Yang X.L., Wang S.Y., Yang N., Zhou W., Wang P., Jiang K., Li S., Song H., Ding X., Chen H. (2019). Oxygen vacancies induced special CO_2_ adsorption modes on Bi_2_MoO_6_ for highly selective conversion to CH_4_. Appl. Catal. B Environ..

[55] Cao Y., Gao Q., Li Q., Jing X., Wang S., Wang W. (2017). Synthesis of 3D porous MoS_2_/g-C_3_N_4_ heterojunction as a high efficiency photocatalyst for boosting H_2_ evolution activity. RSC Adv..

[56] Gordon T.R., Cargnello M., Paik T., Mangolini F., Weber R.T., Fornasiero P., Murray C.B. (2012). Nonaqueous synthesis of TiO_2_ nanocrystals using TiF_4_ to engineer morphology, oxygen vacancy concentration, and photocatalytic activity. J. Am. Chem. Soc..

[57] Gao X., Shang Y., Liu L., Fu F. (2019). Chemisorption-enhanced photocatalytic nitrogen fixation via 2D ultrathin p–n heterojunction AgCl/δ-Bi_2_O_3_ nanosheets. J. Catal..

[58] Zuo C., Tai X., Jiang Z., Liu M., Jiang J., Su Q., Yan X. (2023). S-Scheme 2D/2D Heterojunction of ZnTiO_3_ Nanosheets/Bi_2_WO_6_ Nanosheets with Enhanced Photoelectrocatalytic Activity for Phenol Wastewater under Visible Light. Molecules.

[59] Wu J.J., Xiong L.J., Hu Y.J. (2021). Organic half-metal derived erythroid-like BiVO_4_/hm-C_4_N_3_ Z-Scheme photocatalyst: Reduction sites upgrading and rate-determining step modulation for overall CO_2_ and H_2_O conversion. Appl.Catal. B Environ..

[60] Lu M., Li Q., Zhang C., Fan X., Li L., Dong Y., Chen G., Shi H. (2020). Remarkable photocatalytic activity enhancement of CO_2_ conversion over 2D/2D g-C_3_N_4_/ BiVO_4_ Z-scheme heterojunction promoted by efficient interfacial charge transfer. Carbon.

[61] Yuan Y., Guo R.T., Hong L.F., Lin Z.D., Ji X.Y., Pan W.G. (2022). Fabrication of a dual Sscheme Bi_7_O_9_I_3_/g-C_3_N_4_/Bi_3_O_4_Cl heterojunction with enhanced visible-lightdriven performance for phenol degradation. Chemosphere.

[62] Liu X.Y., Guo R.T., Qin H., Wang Z.Y., Shi X., Pan W.G., Tang J.Y., Jia P.Y., Miao Y.F., Gu J.W. (2019). Fabrication of Bi_2_O_2_(OH)NO_3_/g-C_3_N_4_ nanocomposites for efficient CO_2_ photocatalytic reduction. Colloids Surf. A Physicochem. Eng. Asp..

[63] Bai Y., Ye L.Q., Wang L., Shi X., Wang P.Q., Wei B. (2016). g-C_3_N_4_/Bi_4_O_5_I_2_ heterojunction with I^3−^/I^−^ redox mediator for enhanced photocatalytic CO_2_ conversion. Appl. Catal. B Environ..

[64] Wu J., Li K., Yang S., Song C., Guo X. (2023). In-situ construction of BiOBr/Bi_2_WO_6_ S-scheme heterojunction nanoflowers for highly efficient CO_2_ photoreduction: Regulation of morphology and surface oxygen vacancy. Chem. Eng. J..

[65] Miao Z., Zhang Y., Wang N., Xu P., Wang X. (2022). BiOBr/Bi_2_S_3_ heterojunction with Sscheme structureand oxygen defects: In-situ construction and photocatalytic behavior for reduction of CO_2_ with H_2_O. J. Colloid Interface Sci..

[66] Iizuka K., Wato T., Miseki Y., Saito K., Kudo A. (2011). Photocatalytic Reduction of Carbon Dioxide over Ag Cocatalyst-Loaded ALa_4_Ti_4_O_15_(A=Ca, Sr, and Ba) Using Water as a Reducing Reagent. J. Am. Chem. Soc..

[67] Bai S., Wang X., Hu C., Xie M., Jiang J., Xiong Y. (2014). Two-dimensional g-C_3_N_4_: An ideal platform for examining facet selectivity of metal co-catalysts in photocatalysis. Chem. Commun..

[68] Yang G., Miao W., Yuan Z., Jiang Z., Huang B., Wang P., Chen J. (2018). Bi quantum dots obtained via in situ photodeposition method as a new photocatalytic CO_2_ reduction cocatalyst instead of noble metals: Borrowing redox conversion between Bi_2_O_3_ and Bi. Appl. Catal. B.

[69] Jiang Y., Chen H.Y., Li J.Y., Liao J.F., Zhang H.H., Wang X.D., Kuang D.B. (2020). Z-scheme 2D/2D heterojunction of CsPbBr_3_/Bi_2_WO_6_ for improved photocatalytic CO_2_ reduction. Adv. Funct. Mater..

[70] Yoshino S., Sato K., Yamaguchi Y., Iwase A., Kudo A. (2020). Z-schematic CO_2_ reduction to CO through interparticle electron transfer between SrTiO_3_: Rh of a reducing photocatalyst and BiVO_4_ of a water oxidation photocatalyst under visible light. ACS Appl. Energy Mater..

[71] Xie Z.K., Xu Y.Y., Li D., Meng S.C., Chen M., Jiang D.L. (2021). Covalently Bonded Bi_2_O_3_ Nanosheet/Bi_2_WO_6_ Network Heterostructures for Efficient Photocatalytic CO_2_ Reduction. ACS Appl. Energ. Mater..

[72] Liu Y., Guo J.G., Wang Y., Hao Y.J., Liu R.H., Li F.T. (2021). One-step Synthesis of Defected Bi_2_Al_4_O_7_/β-Bi_2_O_3_ Heterojunctions for Photocatalytic Reduction of CO_2_ to CO. Green. Energy. Environ..

[73] Jin J.R., He T. (2017). Facile synthesis of Bi_2_S_3_ nanoribbons for photocatalytic reduction of CO_2_ into CH_3_OH. Appl. Surf. Sci..

[74] Kong X.Y., Lee W.P.C., Ong W.-J., Chai S.-P., Mohamed A.R. (2016). Oxygen-Deficient BiOBr as a Highly Stable Photocatalyst for Efficient CO_2_ Reduction into Renewable Carbon-Neutral Fuels. ChemCatChem.

[75] Dai W.L., Yu J.J., Deng Y.Q., Hu X., Wang T.Y., Luo X.B. (2017). Facile synthesis of MoS_2_/Bi_2_WO_6_ nanocomposites for enhanced CO_2_ photoreduction activity under visible light irradiation. Appl. Surf. Sci..

[76] Liang L., Lei F.C., Gao S., Sun Y.F., Jiao X.C., Wu J., Qamar S., Xie Y. (2015). Single Unit Cell Bismuth Tungstate Layers Realizing Robust Solar CO_2_ Reduction to Methanol. Angew. Chem. Int. Ed..

[77] Wang J.X., Wei Y., Yang B.J., Wang B., Chen J.Z., Jing H.W. (2019). In situ grown heterojunction of Bi_2_WO_6_/BiOCl for efficient photoelectrocatalytic CO_2_ reduction. J. Catal..

[78] Wei Z.H., Wang Y.F., Li Y.Y., Zhang L., Yao H.C., Li Z.J. (2018). Enhanced photocatalytic CO_2_ reduction activity of Z-scheme CdS/BiVO_4_ nanocomposite with thinner BiVO_4_ nanosheets. J. CO_2_ Util..

[79] Li L., Yang Y., Zhou B., Zhou Y., Zou Z.G. (2022). Dimensional matched ultrathin BiVO_4_/Ti_3_C_2_Tx heterosystem for efficient photocatalytic conversion of CO_2_ to methanol. Mater. Lett..

[80] Mane P., Bagal I.V., Bae H., Kadam A.N., Burungale V., Heo J., Byu S.W., Ha J.S. (2022). Recent trends and outlooks on engineering of BiVO_4_ photoanodes toward efficient photoelectrochemical water splitting and CO_2_ reduction: A comprehensive review. Int. J. Hydrogen Energy.

[81] Bai Y., Yang P., Wang L., Yang B., Xie H.Q., Zhou Y., Ye L.Q. (2019). Ultrathin Bi_4_O_5_Br_2_ nanosheets for selective photocatalytic CO_2_ conversion into CO. Chem. Eng. J..

[82] Duan Z.Y., Zhao X.J., Wei C.W., Chen L.M. (2020). Ag-Bi/BiVO_4_ chain-like hollow microstructures with enhanced photocatalytic activity for CO_2_ conversion. Appl. Catal. A Gen..

[83] Zhao X.Z., Xia Y.G., Li H.P., Wang X., Wei J., Jiao X.L., Chen D.R. (2021). Oxygen vacancy dependent photocatalytic CO_2_ reduction activity in liquid-exfoliated atomically thin BiOCl nanosheets. Appl. Catal. B Environ..

[84] Guan X.S., Zhang X.C., Zhang C.M., Li R., Wang Y.F., Fan C.M. (2020). One-step synthesis of novel Bi/Bi_2_SiO_5_ flower-like composites with highly-efficient CO_2_ photoreduction performance. Compos. Commun..

[85] Huang L., Duan Z.Y., Song Y.Y., Li Q.S., Chen L.M. (2021). BiVO_4_ Microplates with Oxygen Vacancies Decorated with Metallic Cu and Bi Nanoparticles for CO_2_ Photoreduction. Appl. Nano Mater..

[86] Guan X.S., Zhang X.C., Zhang C.M., Li R., Liu J.X., Wang Y.F., Wang Y.W., Fan C.M., Li Z. (2022). In Situ Hydrothermal Synthesis of Metallic Bi Self-Deposited Bi_2_SiO_5_ with Enhanced Photocatalytic CO_2_ Reduction Performance. Sol. RRL.

[87] Wu Y.Z., Yue X.Y., Fan J.J., Hao X.M., Xiang Q.J. (2023). In situ fabrication of plasmonic Bi/CsPbBr_3_ composite photocatalyst toward enhanced photocatalytic CO_2_ reduction. Appl. Surf. Sci..

[88] Chen Q., Ma Y.C., Qi B.N., Zhang T.F., Wang L.L., Shi J.S., Lan X.F. (2021). Z-scheme Bi/AgBiS_2_/P25 for enhanced CO_2_ photoreduction to CH_4_ and CO with photo-themal synergy. Appl. Surf. Sci..

[89] Miao Y.F., Guo R.T., Gu J.W., Liu Y.Z., Wu G.L., Duan C.P., Pan W.G. (2021). Z-Scheme Bi/Bi_2_O_2_CO_3_/Layered Double-Hydroxide Nanosheet Heterojunctions for Photocatalytic CO_2_ Reduction under Visible Light. ACS Appl. Nano Mater..

[90] Zhu J.Y., Li Y.P., Zhao J., Wu Y.S., Li F.T. (2019). Simultaneous Phosphorylation and Bi Modification of BiOBr for Promoting Photocatalytic CO_2_ Reduction. ACS Sustain. Chem. Eng..

[91] Jiang L.S., Li Y., Wu X.Y., Zhang G.K. (2021). Rich oxygen vacancies mediated bismuth oxysulfide crystals towards photocatalytic CO_2_-to-CH_4_ conversion. Sci China Mater..

[92] Zhang Y.Z., Zhi X., Harmer J.R., Xu H.L., Davey K., Ran J.R., Qian S.Z. (2022). Facet-specific Active Surface Regulation of Bi_x_MO_y_ (M=Mo, V, W) Nanosheets for Boosted Photocatalytic CO_2_ reduction. Angew. Chem. Int. Ed..

[93] Sun N.C., Zhou M., Ma X.X., Cheng Z.H., Wu J., Qi Y.F., Sun Y.J., Zhou F.H., Shen Y.X., Lu S.Y. (2022). Self-assembled spherical In_2_O_3_/BiOI heterojunctions for enhanced photocatalytic CO_2_ reduction activity. J. CO_2_ Util..

[94] Li R.J., Luan Q.J., Dong C., Dong W.J., Tang W., Wang G., Lu Y.F. (2021). Light-facilitated structure reconstruction on self-optimized photocatalyst TiO_2_@BiOCl for selectively efficient conversion of CO_2_ to CH_4_. Appl. Catal. B Environ..

[95] Jiang L.S., Wang D.Y., Hu Y., Guo T., Liu C.Y., Liang C., Du W., Li X.Q., Liu W. (2023). Surface-iodination-induced efficient charge separation in bismuth oxysulfide crystals for enhanced photocatalytic CO_2_ conversion. Chem. Eng. J..

[96] Cao S.W., Shen B.J., Tong T., Fu J.W., Yu J.G. (2018). 2D/2D Heterojunction of Ultrathin MXene/Bi_2_WO_6_ Nanosheets for Improved Photocatalytic CO_2_ Reduction. Adv. Funct. Mater..

[97] Zhao D.W., Xuan Y.M., Zhang K., Liu X.L. (2021). Highly Selective Production of Ethanol over Hierarchical Bi@Bi_2_MoO_6_ composite via Bicarbonate-Assisted Photocatalytic CO_2_ Reduction. ChemSusChem.

